# Mumps [muhmps]

**DOI:** 10.3201/eid3202.240153

**Published:** 2026-02

**Authors:** Shaan Mohan, Ahmad Khan

**Affiliations:** University of Leicester Medical School, Leicester, UK

**Keywords:** Mumps, viruses, paramyxovirus, vaccine-preventable diseases

Mumps virus (*Orthorubulavirus parotitidis*) is a paramyxovirus that causes the infective syndrome commonly known as mumps. Mumps often is characterized by a parotitic warping of facial morphology. In obsolete English, *mump* referred to an exaggerated facial expression or grimace.

A possible etymologic origin of mumps suggested by the Oxford English Dictionary is the Old French word *mommer*, which appears to date back to 1263. In the 1400s, that term was used to mean “play[ing] dice in a mask.” Later kindred words include the Middle Dutch term *mommen* (to go about in a mask or disguise) and the regional Norwegian word *mompe* (to chew with a full mouth).

The Centers for Disease Control and Prevention noted that US mumps cases plummeted by >99% after the mumps vaccination program started in 1967, but mumps cases and outbreaks have increased since 2006. Vaccination coverage for kindergartners during the 2023–24 school year decreased to 92.7% for the measles, mumps, and rubella (MMR) vaccine; US kindergartner exemptions from >1 vaccines increased from 3.0% to 3.3%, an uptrend mainly modified by the increase in nonmedical exemptions.

**Figure:**
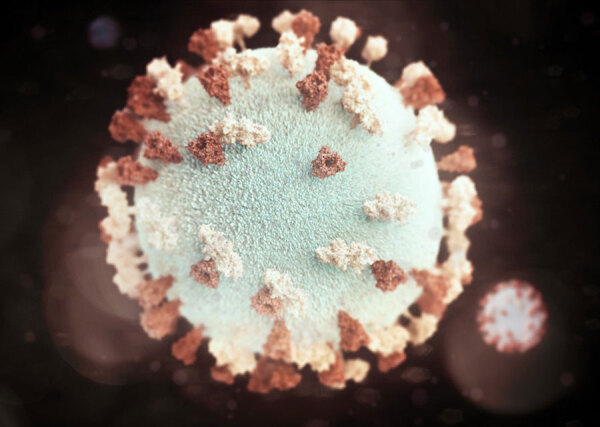
Three-dimensional graphic representation of a spherical-shaped mumps virus particle. Illustrated by Alissa Eckert. Source: Centers for Disease Control and Prevention Public Health Image Library (https://phil.cdc.gov).
